# Teaching Practicum During COVID-19: Pre-Service English Language Teachers’ Professional Identities and Motivation

**DOI:** 10.1177/21582440221119472

**Published:** 2022-09-19

**Authors:** Lee Jin Choi, Mi Yung Park

**Affiliations:** 1 Hongik University, Seoul, South Korea; 2 University of Auckland, Auckland, New Zealand

**Keywords:** pre-service teacher education, teacher identity, teaching practicum, COVID-19, South Korea

## Abstract

As the pandemic has brought in a paradigm shift in the way we educate and interact with our students, it has also had profound impacts on the practicum of pre-service teacher education. Focusing on the case of 14 South Korean student teachers who completed their teaching practicum in Spring 2020, this paper explores how the new form of teaching practicum, triggered by the current outbreak, affected student teachers’ professional development and their views on teaching practice and profession. In particular, it examines the ways by which teaching practicums conducted under unpredictable circumstances negatively or positively affect student teachers’ professional identities as teaching practitioners and their motivation to become a teacher. The findings of this study show that the teaching practicum conducted in times of crisis enabled pre-service English language teachers to develop a positive image toward teachers and teaching profession, and realize their potential as innovative and inspiring teachers in the post COVID-19 era.

## Introduction

The COVID-19 pandemic has disrupted education of more than 1.1 billion students, with 143 countries compelled to close schools. This sudden educational disruption has posed unprecedented challenges to education systems around the world. With the continued spread of COVID-19, many countries have introduced and implemented different forms of emergency remote learning, including distance learning, blended learning, and e-learning, to provide students with a safe learning environment. The impact of the pandemic on education systems has also had profound effects on the practicum of pre-service teacher education (Sepulveda-Escobar & Morrison, 2020). A field-based teaching practicum is generally required to obtain teacher certification; it involves extensive structured classroom observations and supervised teaching practice. Due to school closures and COVID-related confinement, a teaching practicum has shifted from an in-person and field-based practicum to an entirely or partially virtual model. Such a change has compelled educationists to find new ways of conducting teaching practicums in schools (Assunção Flores & Gago, 2020).

As previous research has shown, teaching practicums are one of the most important aspects of teacher education, as they affect professional training and the learning processes of student teachers (Boz & Boz, 2006; Yan & He, 2010), as well as their perceptions of a career in teaching (Hodge et al., 2002; Poulou, 2007). Because pre-service teachers tend to have limited teaching experience prior to their first field-based practicum, teaching practicums are essential for pre-service teachers to “acquire professional knowledge and competences as a teacher” (Hascher et al., 2004, p. 626). In addition, teaching practicums motivate pre-service teachers with regard to teaching and constructing their professional identities as prospective teachers (Deng et al., 2018; Gao & Benson, 2012; Lee & Yuan, 2014; Maaranen & Stenberg, 2020; Sinclair, 2008). While recent studies examine the challenges posed by the pandemic to teacher educators and policymakers (e.g., Cutri et al., 2020; Kidd & Murray, 2020), little attention has been directed toward the experiences of pre-service teachers during teaching practicums conducted all through the pandemic. To prepare for the new post-pandemic world, it is timely to look at the experiences of student teachers as they participated in teaching practicums over the course of the COVID-19 pandemic.

Focusing on the case of 14 South Korean student teachers who completed their teaching practicum in Spring 2020, this study investigates how the new form of teaching practicum, triggered by the COVID-19 outbreak, affected student teachers’ professional development and their views on teaching practice and profession. It focuses on the ways by which teaching practicums conducted under unpredictable circumstances affect student teachers’ professional identities as teaching practitioners and their motivation to become a teacher. Drawing on interviews and reflective journals, the study addresses the following questions:

1. How did a teaching practicum conducted during the COVID-19 outbreak affect student teachers’ perceptions and attitudes toward teaching as a profession?
2. How did a teaching practicum conducted during the COVID-19 outbreak influence how student teachers envisioned themselves as teachers?

## Pre-Service Teacher Professional Identities in Language Teaching

The literature on teacher education for language teaching has successfully demonstrated the importance of teacher professional identities, which that influence teaching practices and the construction of professional selves. Unlike the traditional approach, which tends to view teachers as passive technicians, recent studies have begun to regard teachers as active participants who engage in making sense of their teaching practices and professional positioning in societies (Duff & Uchida, 1997; Tsui, 2007, among many others). Teachers’ professional identities refer to how teachers understand their careers and social positioning and simultaneously reflect on their commitment to a teaching career (Hammerness et al., 2005; Lasky, 2005). Teachers’ identity of being a teacher practitioner is not fixed but rather is a constantly negotiated and reconstructed sense of becoming a professional self while adopting social changes such as accepting educational reforms or receiving teacher education. Developing teacher professional identities is an important part of achieving professional growth and development and can be defined as follows:

[Teacher professional identity] provides a framework for teachers to construct their own ideas of ‘how to be,’ ‘how to act,’ and ‘how to understand’ their work and their place in society. Importantly, teacher identity is not something that is fixed nor is it imposed, rather it is negotiated through experience and the sense that is gained from of that experience. (Sachs, 2005, p. 15)

In pre-service teacher education, a teaching practicum is essential for pre-service teachers to develop their professional identities as teacher practitioners. Recent studies show that teaching practicums can help pre-service language teachers develop positive feelings (Manuel & Hughes, 2006; Watt & Richardson, 2008) and motivate them toward teaching (Caires et al., 2012; Lee & Yuan, 2014). As Lofthouse et al. (2021) point out, a teaching practicum enables pre-service teachers to move from student-mind to teacher-mind and develop their professional identities as prospective teachers. In addition, a teaching practicum provides pre-service teachers with an opportunity to actively participate in the process of negotiating and developing their professional identities as teachers (Deng et al., 2018; Gao & Benson, 2012; Poulou, 2007). Moreover, the development of a teacher identity is important for pre-service teachers because their professional identities and degree of motivation can greatly affect their career choices in teaching and their satisfaction with their decisional status (Dörnyei & Ushioda, 2011; Richardson, 2003; Sinclair, 2008). As Lee and Yuan (2014) have stated, “a teaching practicum sets the stage for success or failure in student teaching and a student teacher’s future in education” (p. 57).

Identity development is central to pre-service teachers’ professional development. Because teacher professional identity is rooted in core beliefs an individual teacher holds toward teaching, it is closely related to their motivation and their career decision-making and career development (Hargreaves et al., 2006). Bennett (2015), who examined pre-service teachers’ motivation and professional identities, argued that developing their sense of self as a teacher and being able to visualize themselves as capable teachers are closely related to their degree of career motivation and life goal aspirations. In teacher education programs, including teaching practicums, pre-service teachers constantly engage in a dynamic process of developing their own sense of self as a teacher in relation to professional environments and demands. This process of positioning oneself in a teaching profession enables pre-service teachers to better understand themselves as prospective teachers, as well as their perceived suitability and desire for a teaching career. Previous studies have shown that developing a positive perception of teaching and envisioning themselves as effective teachers in the future are important elements in pre-service teachers’ development toward becoming good teachers (e.g., Connelly & Clandinin, 1999; Danielewicz, 2001; Steffy et al., 2000).

## The Impact of COVID-19 on the South Korean Education System and Its Teaching Practicum

Like many other countries in the world, the South Korean education system has been markedly affected by the COVID-19 outbreak. South Korea’s academic year normally starts in March, but the opening of schools was postponed four times between February and March 2020. Amid growing concerns regarding the community spread of COVID-19, the Ministry of Education announced that schools would begin the spring semester online and then consider moving toward blended learning, a hybrid form of in-person and online classes (Ministry of Education, 2020). South Korea resumed schools online in April 2020, starting with high- and middle-school seniors on April 9, followed later the same month by other grades. To facilitate online learning during the suspension of in-person learning, the Korean Education Broadcasting System (EBS) and the Korea Education and Research Information Service (KERIS) provided online learning material, including video-recorded lectures, digital textbooks and worksheets, and online teaching-learning platforms such as the EBS online class.

Schools reopened for in-person instruction in mid-May by taking everyday preventive measures related to the COVID-19 pandemic, including the mandated use of face masks, checking students’ and staff’s body temperature twice a day, and keeping one-third of all windows open when air conditioners were on. To minimize the risk of the spread of the disease, schools also followed strict physical distancing measures, including scattered attendance. For example, most Korean elementary schools use a blended learning approach by which students attend schools 1 day a week and attend online classes at home 4 days a week (Table 1).

**Table 1.** School Terms and Important Dates of Reopening for Spring 2020 in South Korea.

School reopening with online classes only	School reopening with a combination of online and off-line classes	Teaching practicum schedule
April 9th: reopening for high- and middle-school seniors	May 20th: reopening for high school seniors	May 18th to May 31st: online teaching practicum
April 16th: reopening for 4th to 11th graders	May 27th: reopening for 11th graders, middle school seniors, and first to second graders	June 1st to June 14th: field-based teaching practicum
April 20th: reopening for first to third graders	June 3rd: third–fourth graders	
	June 8th: fifth–seventh graders	

The teaching practicum in South Korea is an important component of pre-service teacher education. A 4-week, full-time teaching practicum that consists of 60 hours of field-based supervised teaching experiences is one of the major requirements for obtaining teacher certification in South Korea. This teaching practicum is generally attended by seniors in colleges for teachers during the spring semester of their final year in school. In 2020, the practicum was initially scheduled starting from mid-April, but it was postponed and held in May and June instead. In following guidelines from the Ministry of Education, the duration of the field-based practicum was also reduced to 2 weeks, combined with 2 weeks of an online teaching practicum, which included 30 hours of attending an online teacher education program provided by the National Education Training Institute. Some issues addressed in the program include “effective communication strategies for teachers,” “teaching with multimedia,” “creating digital educational resources,” and “dealing with school violence.” Because this practicum took place shortly after schools reopened for in-person classes, the field-based practicum mostly involved the observation of online classes and the creation of online teaching materials, with few opportunities to interact with students in on-site, face-to-face classrooms.

## Study

### Context and Participants

The study assessed 14 pre-service teachers majoring in English education. They participated in a teaching practicum in Spring 2020. At the time of data collection, all of them were enrolled in the course, which was designed to prepare pre-service teachers to undertake their first teaching practicum. A total of 17 students who enrolled in the course were invited to participate in the research, and 14 of them (approximately 82%) voluntarily participated. The institutional review board approved the study and informed consents were obtained from all the participants (7002340-202005-HR-007). The course was taught by the first author who supervised and guided pre-service teachers through the process of preparing, conducting, and reflecting on the teaching practicum. Like other South Korean pre-service teachers, none of the participants had any teaching experience in public school settings prior to participation in this practicum. The pre-service teachers were randomly assigned to either middle schools or high schools for the practicum; seven participants were placed in middle schools and the other seven were placed in high schools.

While a teaching practicum generally offers pre-service teachers an opportunity to have practical experiences with students and teachers in on-site classroom settings, the teaching practicum conducted in Spring 2020 involved a combination of online and offline teaching. Although the pre-service teachers were physically present in schools during the 2-week practicum, the observation of classes and teaching practices were mostly carried out online, with minimal contact with students. The experiences that pre-service teachers had during the practicum differed widely, depending upon the schools and regions (see Table 2). About 8 out of the 14 pre-service teachers were given an opportunity to teach in person: four of them taught students in person, while the other four taught peer pre-service teachers in person. Thirteen pre-service teachers were asked to create online teaching materials for one or two lessons but did not have an opportunity to use them in either online or offline classes. None of them had an opportunity to teach online classes; however, they gained extensive experience with observing online classes taught by supervised teachers for a total of between 5 and 20 hours. All of them had opportunities to assist supervised teachers in utilizing online educational platforms such as EBS online classrooms to provide class-related materials and check students’ assignments and progress.

**Table 2.** Participants and Their Teaching Experiences During the Practicum.

Name	Gender	School	Teaching practice
Minsoo	Male	High school	Teaching in-person: 0 lesson
			Teaching online: 0
			Creating online class materials: 1 lesson
Jeongmin	Male	Middle school	Teaching in-person: 1 lesson (with peer teachers)
			Teaching online: 0
			Creating online class materials: 2 lessons
Yeonju	Female	Middle school	Teaching in-person: 1 lesson (with peer teachers)
			Teaching online: 0
			Creating online class materials: 2 lessons
Sangin	Male	High school	Teaching in-person: 0
			Teaching online: 0
			Creating online class materials: 1 lesson
Homin	Male	High school	Teaching in-person: 0
			Teaching online: 0
			Creating online class materials: 1 lesson
Eunsu	Female	Middle school	Teaching in-person: 1 lesson (with peer teachers)
			Teaching online: 0
			Creating online class materials: 2 lessons
Seongho	Male	Middle school	Teaching in-person: 1 lesson (with peer teachers)
			Teaching online: 0
			Creating online class materials: 2 lessons
Ayoung	Female	Middle school	Teaching in-person: 1 lesson (with students)
			Teaching online: 0
			Creating online class materials: 1 lesson
Jihoon	Male	High school	Teaching in-person: 0
			Teaching online: 0
			Creating online class materials: 1 lesson
Sohee	Female	High school	Teaching in-person: 3 lessons (with students)
			Teaching online: 0
			Creating online class materials: 0 lesson
Sumin	Female	High school	Teaching in-person: 1 lesson (with students)
			Teaching online: 0
			Creating online class materials: 1 lesson
Eunbum	Male	Middle school	Teaching in-person: 1 lesson (with students)
			Teaching online: 0
			Creating online class materials: 1 lesson
Byeonghee	Male	High school	Teaching in-person: 0
			Teaching online: 0
			Creating online class materials: 1 lesson
Yumin	Female	High school	Teaching in-person: 0 lesson
			Teaching online: 0
			Creating online class materials: 2 lessons

### Data Collection and Analysis

Since this study aims to explore pre-service teachers’ professional development and identity construction, we employed a qualitative approach to collect and analyze data. The collected data includes qualitative interviews with participants done before and after the teaching practicum, reflective journals written by each participant, and classroom observations. Since qualitative interviews allow interviewees to share their experiences and perspectives in their own words (Rubin & Rubin, 2005), such interviews were conducted with participants before and after the teaching practicum occurred. All the interviews were conducted online by the first author using a video-conferencing tool due to growing concerns related to community spread of the COVID-19 virus. Each interview lasted from 20 to 45 minutes, and all the interviews were conducted in Korean.

In addition, we collected 84 entries (approximately 160 pages) in reflective journals, wherein the participants recorded and reflected upon various experiences encountered during the teaching sessions. Because reflective journals show how teachers develop their understanding and practice of education (Lee, 2008; Ray & Coutler, 2008), our analysis of participants’ reflective journals enabled us to better understand their experiences and professional development. Online classroom observations were made by the researchers to fully conceptualize their teaching experiences during the practicum.

Bogdan and Biklen (2007) describe qualitative data analysis as an iterative process of “working with data, organizing it, breaking it into manageable units, synthesizing it, searching for patterns, discovering what is important and what is to be learned, and deciding what you will tell others” (p. 145). Using constant comparison analysis and grounded theory, the data collected were qualitatively analyzed to derive and identify core categories and emerging themes. The first step in data analysis was to transcribe data and familiarize ourselves with it by reading and annotating the transcripts. This involved examining data to note recurring patterns and ideas to guide the analysis and identify certain relationships, such as similarities and differences in pre-service teachers’ narratives of their experiences. The second step involved sorting the data through line-by-line coding and identifying notable segments of data that were closely related to research questions. The third step consisted of grouping the initial codes to generate emerging themes, which then were constantly compared and examined for connections (Strauss & Corbin, 1994). Memo writing was used throughout the analysis to document and reflect on emerging ideas, questions, and themes. The data presented in this paper are representative of the entire data set.

## Teaching Practicum Under the Effects of COVID-19: Pre-Service Teachers’ Perceptions About Teaching

Before starting their field-based teaching practicum, many pre-service teachers in this study expressed their concerns about the uncertain and unstable nature of the practicum. As the reopening of schools raised concerns about the entry and spread of COVID-19 among students and staff, most schools were unsure about what types of practical experiences they would allow pre-service teachers to have during the practicums. During the interviews conducted before the practicum, these teachers all exhibited frustration and uncertainty regarding their capability to carry out the teaching practicum. In addition, they worried about whether the experiences they would have during the practicum would help them achieve the level of professional development they sought. In addition, witnessing many in-service teachers struggling with unpredictable situations, more than half of the participants expressed doubts about teaching as a profession. The following quotes, taken from interviews conducted prior to the practicum, are representative in this regard:

I always wanted to be a teacher and sort of looked forward to participating in a teaching practicum. But I became very skeptical about teaching career. It seems like teachers are forced to work so hard during the current outbreak without getting any adequate supports. (Eunsu, Interview #1)

I don’t want to participate in a teaching practicum in this uncertain time. And I don’t think that I would like to be a teacher anymore. Over the course of this outbreak, teachers, like doctors, work hard to actively respond to the crisis and try to keep their students safe. But unlike doctors who are treated as heroes, teachers are to be blames for being incompetent and failing to provide quality education online. (Jihoon, Interview #1)

I always thought that teaching profession is a very safe and secure job. But watching teachers struggling to switch to online classes and checking students’ temperature each class, I realized that it was not the case. Teachers are expected to keep adapting to the ever changing educational environments and going the extra mile for their students. I think the salary is way low for the effort and responsibilities. (Byeonghee, Interview #1)

Despite the teachers’ reported initial frustrations and anxieties, both the interviews conducted after the practicum and reflective journals clearly show that the teaching practicum provided them with an opportunity to develop positive feelings about teaching, and to fully understand and appreciate the role of teacher practitioners in times of crisis. Because the field-based teaching practicum started shortly after schools were reopened for in-person instruction, pre-service teachers were able to closely observe the entire process by which in-service teachers design a blended learning arrangement and learn to utilize new online learning-teaching platforms, as provided by the Ministry of Education. They also learned to manage a hybrid form of in-person and online classes while simultaneously attending to students’ health and well-being. In their reflective journals, the participating teachers described specific incidents or situations that made them highly respect other teachers for the dedication they showed toward their students. Some excerpts from the reflective journals of the students are provided below:

The teaching practicum made me realize that teaching is an incredibly challenging but yet a most rewarding job. I was very impressed by how dedicated the teachers are towards students and how much positive differences they make. Although wearing masks all day long and constantly checking everyone’s temperature and body condition puts a lot of stress on teachers, they always try to be positive and attentive to students who need extra support. . . Experiencing community spread, they must be also scared and anxious; however, they keep their students’ well-being first and center. I feel so lucky to have such great teachers as a part of our society and would love to be one of them in the future. (Sohee, Journal entry)

I did not expect much, but the experiences I had were incredible. . . During the practicum, I watched how hard teachers try to design and offer effective blended learning classes and monitor and support each student’s needs. At the same time, they took extraordinary efforts to stay in touch with their students and parents. As a pre-service teacher, I did my best to assist my supervising teacher and provide students individualized help. I did not have much chance to interact with students in person; however, constantly monitoring students’ progress and interacting with students using online teaching platforms made me realize how important the role of teachers is, to encourage students to stay connected with the school under these frightening times. (Yeonju, Journal entry)

Similar accounts could also be found in narratives of other pre-service teachers about the teaching practicum, wherein they describe teachers as “great” (*daedanhan*), “respectable” (*jongyeongseuleoun*), and “heroic” (*yeongunggateun*). During interviews conducted after the practicum, all the pre-service teachers stated that the field-based teaching practicum enabled them to have more positive feelings about teaching, as well as more respect for teachers and a greater appreciation for the challenging nature of their job. They explained that their experiences of observing and helping in-service teachers, who were committed to ensuring that all students had the guidance, skills, and support needed to stay on track and continue to be engaged in learning in times of crisis, were inspirational and motivating. Moreover, many participants stated that such experiences gave them an opportunity to develop positive feelings about teaching, assume responsibilities as teachers, and enabled them to move from a student-mind to a teacher-mind (c.f., Ishihara, 2005; Lofthouse et al., 2021).

## Teaching Practicum Under COVID-19: Pre-Service Teachers’ Envisioning of Possible Selves as Teachers

The current COVID-19 outbreak brought online learning to the forefront of education (The World Bank Brief, 2020). As one of the leading countries that reported COVID-19 cases in the earlier phase of the epidemic (Ministry of Education, 2020), South Korea largely promoted blended learning in order to continue education amid the pandemic. Many Korean universities began implementing a set of blended learning practices in late April of 2020, and most K-12 schools did the same starting in May of that year. The teaching practicum conducted in Spring 2020 included extensive experience with observing online classes; creating online teaching materials, including online class videos and worksheets; providing feedback to students on their progress in online learning; and interacting with students online to address their concerns. Such experiences were quite new to all the participants, since most of the courses that they had taken in the university as a part of the teacher education curriculum and their micro-teaching demonstrations were mostly based on on-site, in-person classroom settings.

In both interviews conducted after the practicum, plus the information recorded in reflective journals, many participants stated that they initially found it challenging to learn and utilize new digital technologies to design and develop online classes during the practicum. It is noteworthy that they attributed their challenges mainly to the difficulty of making their English classes more interactive and authentic, rather than having to attend to technology-related issues. When we asked them whether they had any difficulty learning and using digital technologies, 12 of the 14 participants replied that they were familiar with digital technologies, devices, and applications being used for blended learning classes in schools. Despite their initial difficulties, 10 of the 14 participants indicated that they enjoyed learning new technologies and participating in designing and developing blended learning classes. They also pointed out that their familiarity with digital technologies and devices enabled them to provide better and more effective blended learning experiences for students.

Excerpts from a journal entry and an interview are quoted below:

In my school, none of English teachers had implemented blended learning before. They form a professional community to discuss how to make blended learning more meaningful and engaging. I learned a lot from their discussions, and at the same time, I put forward some useful suggestions, such as how to utilise YouTube Studio for individual student assignments. I like to try out new technology and devices, and the practicum made me realize that this could be my strong point. I realized that I could be a super innovative and cool English teacher in the post COVID-19 era. (Seongho, Journal entry)

I was fortunate enough to teach in an in-person class but with strict physical distancing rules including no verbal interaction among students. At first, I did not know what to do because English classes are all about being student-centred and interactive. After getting advice from you [the first author] via email and sharing my concerns with my supervising teacher, I started to think more creatively—“I have 25 tablets and the WiFi in the class. What can I do to make my class interactive and engaging?” I designed the class schedule such that the students could share their opinions via Google forms that were screen-mirrored on the main screen, as well as checked their learning via an online learning game and engaged them in online collaborative writing as part of a group project. Students just loved it! Although several students had some minor technology-related issues, all of them actively participated in the online discussions and activities. I was so proud of myself. I am far-removed from tech-savvy millennials, so I never thought of myself as enjoying a digital learning environment. Nevertheless, I really enjoyed the experience and will definitely be able to do better next time. (Eunbum, Interview #2)

Both of the above excerpts show that the experiences that pre-service teachers had during the practicum positively affected their motivation toward teaching and enabled them to see their potential as creative English teachers in a post COVID-19 era. In addition, some participants pointed out that the practicum was designed in such a way that they better understood what a career in teaching would mean to them. Recent studies allege that many pre-service teachers in South Korea have pursued a teaching career not because they find it rewarding and motivating, but because it is stable and secure—factors that could negatively affect pre-service teachers’ professional development and identity formation (Han, 2012; Lee, 2014). During the interview, four participants stated that their experiences of observing and engaging in blended learning enabled them to understand a teaching career as dynamic, innovative, and energetic. The following interview conducted after the practicum illustrates how Ayoung changed her perception of teaching and her related career choice:

Ayoung: I never wanted to be a teacher, but after participating in the practicum I am considering becoming a teacher.

Researcher: Can I ask you why?

Ayoung: My parents always said that teaching is the most secure job. You know, being secure means boring, boring and boring! But I learned that teaching is not about doing the same job for my entire life but constantly learning and trying new teaching methods and technologies to meet the needs of the fast-changing world.

Researcher: What triggered your perspective toward teaching?

Ayoung: The teachers who I met during the practicum of course changed my perspectives. They would just allow students watch EBS televised lectures instead of creating their own online classes. However, they put students first and worked together to design blended learning practices suitable for their students’ needs. They made me realize that being a teacher means making a difference in students’ lives and engaging them in the process of constant self-development. I love it!

(Interview with Ayoung, July 2020)

As shown in the above excerpt, the teaching practicum conducted during this particular time of crisis provided pre-service teachers with the opportunity to better understand the dynamic nature of the teaching practice, which would help them develop a positive image about teachers and the teaching profession, and to motivate them to choose teaching as a career.

## Discussion and Conclusion

With the rapid spread of COVID-19, educational systems across the world have faced unprecedented challenges to provide students with viable continuing learning opportunities while supporting quality distance learning. In the current circumstances, teacher educators struggle to provide pre-service teachers with sufficient but safe practical experiences that adequately prepare them for the teaching profession (Assunção Flores & Gago, 2020). With the situation changing daily under the various influences of the COVID pandemic, many teacher education programs were forced to shift from a field-based practicum to an entirely or partially virtual teaching practicum model. These new forms of teaching practicums caused by the current outbreak have raised various concerns among teacher educators and school administrators regarding the nature of experiences that pre-service teachers might have in schools, and whether these experiences would help them achieve a satisfactory degree of professional development.

The findings of this study show that a teaching practicum conducted in such a time of crisis enabled pre-service teachers to develop a positive image of teachers and the teaching profession, and realize their potential as innovative and inspiring teachers in a post COVID-19 era. The findings also demonstrate that pre-service teachers’ experiences in closely observing the entire process of how teachers and school administrators cope with these new challenges stemming from the pandemic caused pre-service teachers to positively shift their understanding of the role of teachers while helping them construct positive professional identities as prospective teachers. As Assunção Flores and Gago (2020) note, a teaching practicum in times of crisis can provide pre-service teachers with diverse and innovative experiences. In addition, a teaching practicum is an important site for identity formation, one in which pre-service teachers can explore and develop their sense of self as a teacher while strengthening positive emotions such as self-confidence and overall job satisfaction (Bennett, 2015; Darling-Hammond, 2017).

The findings of the study also provide some practical implications and suggestions for teacher educators. First, it is important to create a more sustainable partnership between a school and university to provide pre-service teachers with more flexible and adaptable teaching experiences. A sustainable school-university partnership could allow all the actors—school administrators, practitioners, teacher educators and pre-service teachers—to become involved in the decision making process for a complex situation such as ongoing COVID-19 pandemic (c.f., Heinz & Fleming, 2019). This could decrease pre-service teachers’ initial levels of anxiety and uncertainty about teaching and participating in the teaching practicum, as reported by many participants prior to undertaking the practicum. Second, teacher education programs should provide pre-service teachers with opportunities to explore and develop their professional identities before, during, and after the teaching practicum. As the findings indicate, pre-service teachers’ professional identities are not fixed but fluid, constantly changing over the course of teacher education. To help pre-service teachers actively participate in the process of their professional identity (re)construction, it is essential that teacher education programs provide pre-service teachers space and forums through which they can navigate their respective paths based on their experiences and develop their voices as prospective teachers.
